# Effects of spermidine supplementation on cognition and biomarkers in older adults with subjective cognitive decline (SmartAge)—study protocol for a randomized controlled trial

**DOI:** 10.1186/s13195-019-0484-1

**Published:** 2019-05-01

**Authors:** Miranka Wirth, Claudia Schwarz, Gloria Benson, Nora Horn, Ralph Buchert, Catharina Lange, Theresa Köbe, Stefan Hetzer, Marta Maglione, Eva Michael, Stefanie Märschenz, Knut Mai, Ute Kopp, Dietmar Schmitz, Ulrike Grittner, Stephan J. Sigrist, Slaven Stekovic, Frank Madeo, Agnes Flöel

**Affiliations:** 1 Charité – Universitätsmedizin Berlin, Corporate Member of Freie Universität Berlin, Humboldt-Universität zu Berlin, and Berlin Institute of Health, Klinik und Hochschulambulanz für Neurologie, Berlin, Germany; 2 Charité – Universitätsmedizin Berlin, Corporate Member of Freie Universität Berlin, Humboldt-Universität zu Berlin, and Berlin Institute of Health, NeuroCure Clinical Research Center, Berlin, Germany; 3 Charité – Universitätsmedizin Berlin, Corporate Member of Freie Universität Berlin, Humboldt-Universität zu Berlin, and Berlin Institute of Health, Center for Stroke Research Berlin, Berlin, Germany; 4 German Center for Neurodegenerative Diseases (DZNE), Dresden, Germany; 5 Charité - Universitätsmedizin Berlin, Corporate Member of Freie Universität Berlin, Humboldt-Universität zu Berlin, and Berlin Institute of Health, Department of Nuclear Medicine, Berlin, Germany; 6 University Hospital Hamburg-Eppendorf, Department of Diagnostic and Interventional Radiology and Nuclear Medicine, Hamburg, Germany; 7 Douglas Mental Health University Institute, Studies on Prevention of Alzheimer’s Disease (StOP-AD) Centre, Montreal, Quebec, Canada; 8 Department of Psychiatry, McGill University, Montreal, Quebec, Canada; 9 Charité – Universitätsmedizin Berlin, Corporate Member of Freie Universität Berlin, Humboldt-Universität zu Berlin, and Berlin Institute of Health, Berlin Center for Advanced Neuroimaging, Berlin, Germany; 10 Bernstein Center for Computational Neuroscience, Berlin, Germany; 11 Institute for Biology/Genetics, Freie Universität Berlin, Berlin, Germany; 12 Charité - Universitätsmedizin Berlin, Corporate Member of Freie Universität Berlin, Humboldt-Universität zu Berlin, and Berlin Institute of Health, Department of Endocrinology & Metabolism, Berlin, Germany; 13 Charité-Center for Cardiovascular Research (CCR), Berlin, Germany; 14 German Center for Neurodegenerative Diseases (DZNE), Berlin, Germany; 15 Charité – Universitätsmedizin Berlin, Corporate Member of Freie Universität Berlin, Humboldt-Universität zu Berlin, and Berlin Institute of Health, Institute of Biometry and Clinical Epidemiology, Berlin, Germany; 16 Berlin Institute of Health (BIH), Berlin, Germany; 17 Institute of Molecular Biosciences, University of Graz, NAWI Graz, Graz, Austria; 18 BioTechMed, Graz, Austria; 19 Department of Neurology, University Medicine Greifswald, Greifswald, Germany; 20 German Center for Neurodegenerative Diseases (DZNE), Rostock/Greifswald, Rostock, Germany

**Keywords:** Dietary supplementation, Polyamines, Aging, Memory, Health, Autophagy, Nutrition

## Abstract

**Background:**

Given the global increase in the aging population and age-related diseases, the promotion of healthy aging is one of the most crucial public health issues. This trial aims to contribute to the establishment of effective approaches to promote cognitive and brain health in older individuals with subjective cognitive decline (SCD). Presence of SCD is known to increase the risk of objective cognitive decline and progression to dementia due to Alzheimer’s disease. Therefore, it is our primary goal to determine whether spermidine supplementation has a positive impact on memory performance in this at-risk group, as compared with placebo. The secondary goal is to examine the effects of spermidine intake on other neuropsychological, behavioral, and physiological parameters.

**Methods:**

The SmartAge trial is a monocentric, randomized, double-blind, placebo-controlled phase IIb trial. The study will investigate 12 months of intervention with spermidine-based nutritional supplementation (target intervention) compared with 12 months of placebo intake (control intervention). We plan to recruit 100 cognitively normal older individuals with SCD from memory clinics, neurologists and general practitioners in private practice, and the general population. Participants will be allocated to one of the two study arms using blockwise randomization stratified by age and sex with a 1:1 allocation ratio. The primary outcome is the change in memory performance between baseline and post-intervention visits (12 months after baseline). Secondary outcomes include the change in memory performance from baseline to follow-up assessment (18 months after baseline), as well as changes in neurocognitive, behavioral, and physiological parameters (including blood and neuroimaging biomarkers), assessed at baseline and post-intervention.

**Discussion:**

The SmartAge trial aims to provide evidence of the impact of spermidine supplementation on memory performance in older individuals with SCD. In addition, we will identify possible neurophysiological mechanisms of action underlying the anticipated cognitive benefits. Overall, this trial will contribute to the establishment of nutrition intervention in the prevention of Alzheimer’s disease.

**Trial registration:**

ClinicalTrials.gov, NCT03094546. Registered 29 March 2017—retrospectively registered.

**Protocol version:**

Based on EA1/250/16 version 1.5

## Background

Longer life expectancy has led to a growth of the older population, with individuals aged 60 and older expected to reach about 20% of the population in high-income countries [1]. This demographic change is associated with increased rates of age-related diseases, such as dementia due to Alzheimer’s disease (AD). The most prevalent form of late-onset dementia is expected to triple by the year 2050 [2], which will pose a high social and economic impact on patients, caregivers, and society in general. Importantly, around one third of these cases worldwide are attributed to potentially modifiable risk factors [3]. The development of effective strategies that help to prevent age- and disease-related worsening of brain structure and function may thus provide significant benefits for society and health-care systems.

### Subjective cognitive decline: a target group for early intervention

Subtle neuropathological alterations related to AD are suggested to start decades before the onset of clinical symptoms [4, 5]. One early sign of pathological brain aging is the manifestation of subjective cognitive decline (SCD). In cognitively unimpaired older individuals, the presence of SCD is associated with a higher risk for objective cognitive decline and clinical progression to symptomatic disease stages [6–9]. Moreover, individuals with SCD harbor increased β-amyloid (Aβ) deposition [10], gray matter volume reduction [11–13], and neural dysfunction [14] in brain regions typically affected in AD. Thus, SCD has been conceptualized to occur at a late preclinical stage of AD [15], where aberrant brain changes are present [16] in the absence of objective cognitive impairment. This at-risk group is recognized as an eligible target population for early intervention strategies [15, 17], aiming to protect against neuropathological alterations, to restore functional and structural brain health, and to maintain cognitive abilities as long as possible.

### Spermidine and its implication in healthy aging

Healthy lifestyle behaviors, including physical activity, cognitive attainment, and a healthy diet, are proposed to protect against age- and disease-related brain changes and, thereby, preserve cognitive functioning [18–22]. Caloric restriction, among others, appears to be effective to improve memory performance in the elderly [23] and induce favorable neural changes in the hippocampal network [24]. Novel candidate substances proposed to mimic such beneficial effects in aging organisms are natural polyamines, in particular, spermidine and spermine [25, 26]. Organic compounds play an important role in the maintenance of basic cellular functions like cell growth, survival, and proliferation [27]. Beyond these “microscopic” actions, there is an indication that polyamines influence “macroscopic” systems underlying learning and memory as well as age-related changes of this cognitive function. In rodent models, polyamine levels in the hippocampus are shown to be associated with memory retrieval and formation [28] and change with age in certain brain areas, including medial-temporal memory structures [29]. Lastly, the reduction of spermidine in the brain of aged flies is paralleled by memory decline [30].

Given these observational findings, it has been hypothesized that external supply of polyamines may protect against age-related memory loss. Indeed, a first study in aging fruit flies showed that spermidine-rich diet restored endogenous spermidine levels and thereby rescued memory performance [30]. This beneficial effect of spermidine intake appears to be mediated by several protective pathways [31]. For example, spermidine may act through autophagy to regulate synaptic transmission/plasticity [32] and clear cellular “waste” including pathogenic protein aggregates [30]. Nutritional spermidine is also associated with a number of cardio-protective [33] and anti-inflammatory [34] actions, which may help to preserve higher-order brain functions. Overall, these findings in aging model organisms have suggested a promising role of spermidine in the promotion of brain and cognitive health.

### Spermidine supplementation in older adults with SCD: initial evidence

Spermidine supplementation is thus proposed to open a new avenue in the protection and restoration of memory abilities in higher age. This expected benefit is of particular need in older individuals at risk for the development of dementia. It is, however, unknown whether the memory-promoting effect of spermidine is detectable in humans and to what extent this effect may be attributed to the influence of spermidine on biomarkers of healthy aging. An initial study showed that polyamine-enriched diet over 2 months increased blood spermidine levels in healthy middle-aged men [35]. Our group has conducted a first 3-month phase IIa trial with nutritional spermidine in 30 older adults with SCD (ClinicalTrials.gov identifier NCT02755246). Trial outcomes demonstrated high compliance, tolerance, and safety profiles as well as preliminary efficacy of the administered spermidine-rich plant extract [36, 37]. Specifically, we found a moderate enhancement of memory performance, measured using the mnemonic similarity task (MST) [38], in the spermidine-treated group compared with placebo intervention. This computer-based task is a sensitive measure of subtle cognitive changes induced by targeted interventions in the memory system [39]. In the pilot trial, we did not detect intervention effects on standard neuropsychological tests of memory or executive functions [37]. At this point, longer-term intervention studies with sufficient sample size are required to validate the therapeutic potential of nutritional spermidine against memory loss in older individuals and delineate possible neurophysiological mechanisms of action.

### Choice of comparator

A placebo comparator will be implemented in this study, in correspondence with our previous trial [36]. The present trial will use microcrystalline cellulose as a comparator condition. Beside randomization and double blinding, placebo-controlled trials allow to minimize the risk of bias and to maximize the verification of the effect of the verum intervention [40]. Our placebo capsules will be identical to the verum intervention in shape, color, taste, and smell, but contain no active ingredients. The World Health Organization (WHO) and the US Food and Drug Administration (FDA) recognize that the use of cellulose as a food additive is safe and well tolerated in animal models and humans.

### Objective and purpose of the SmartAge trial

We will conduct a randomized controlled trial with a 12-month spermidine supplementation in cognitively unimpaired older individuals with SCD (*n* = 100). The primary objective of the SmartAge trial is to provide evidence of a beneficial impact of nutritional spermidine on memory performance (primary outcome) at the end of the intervention, as compared with placebo. Second, we aim to examine whether spermidine intake has positive effects on memory performance after an additional 6-month follow-up period without further supplementation as well as on other age-relevant cognitive domains, lifestyle behaviors, psycho-affective characteristics, and perceived quality of life. This data will help to estimate potential benefits of nutrition intervention on well-being and everyday life. Third, this trial will identify possible mechanisms of action underlying the proposed spermidine-associated benefits on cognition using indicators of autophagy, blood-based biomarkers, and neuroimaging parameters of brain structure and function. Finally, the study will assess potential moderators of the intervention effect, such as age-related neuropathologies as well as genetic polymorphisms. Overall, the SmartAge trial aims to establish a significant milestone in the implementation of early intervention strategies in older individuals at risk of dementia due to AD.

## Methods: participants, intervention, and outcomes

### Trial design and setting

This is a monocentric, randomized, double-blind, placebo-controlled phase IIb trial, carried out at the NeuroCure Clinical Research Center, Charité – Universitätsmedizin Berlin.

The trial includes 12 months of intervention with spermidine supplementation (target intervention) compared with 12 months of placebo intake (control intervention). The trial will compare outcomes of the two intervention groups, with participants randomized to one of the two study arms. Randomization is performed blockwise with a 1:1 allocation ratio. The SmartAge trial has been approved by the responsible Institutional Review Board and will be carried out in compliance with institutional ethical standards and the Declaration of Helsinki.

### Eligibility criteria

The main inclusion criteria for potential participants in the SmartAge study are:

1. Age, 60–90 years
2. Presence of SCD in accordance with research criteria, recommended by the international SCD-I working group for studies on SCD [15]: the expression of subjective cognitive complaints for at least 6 months and associated concerns (worries), affirmation to consult and/or previous consultation of a doctor due to these symptoms, normal cognitive performance, and no restrictions on activities of daily living
3. Ability to provide written informed consent
4. Health insurance coverage to clarify possible incidental findings

We will exclude potential participants in case one or more of the following criteria are present:

1. Dementia, according to the Diagnostic and Statistical Manual of Mental Disorders, 4th Edition (DSM-IV) [41]
2. Mild cognitive impairment (MCI), according to clinical diagnostic criteria [42]
3. Severe or untreated medical disorders (advanced cardiac or respiratory disease, severe liver, kidney or metabolic diseases, untreated thyroid dysfunctions or untreated diabetes mellitus), psychiatric disorders (untreated depression, psychosis) or neurological disorders (epilepsy, clinically manifest stroke)
4. Malignancies currently or as indicated by medical history (exception: basalioma)
5. Drug abuse or alcohol dependency
6. Current polyamine substitution and/or participation in respective intervention studies
7. Known intolerance or allergies to wheat germs, gluten or histamine
8. Contraindications to imaging techniques: claustrophobia, metallic implants (e.g., intracranial metal clips), electronic devices (e.g., cardiac pacemakers), or permanent tattoos
9. With regard to positron emission tomography (PET) assessments (optional consent): participation in another study with the use of ionizing radiation within the last 3 months
10. With regard to muscle biopsy assessments (optional consent): allergy or intolerance to the local anesthetic (lidocaine), coagulation disorders, current therapy with antiplatelet or anticoagulation drugs (e.g., clopidogrel, aspirin, vitamin K antagonist, new oral anticoagulant drugs) or steroids, current thrombosis, or other severe diseases of the lower extremities that precludes muscle biopsy

In case written informed consent is provided and all eligibility criteria are met, participants will be included in the study.

### Intervention

Participants will receive nutrition intervention over 12 months with either spermidine or placebo supplementation. The dietary supplement used in the target intervention is a polyamine-rich plant extract [36], produced using an extraction method developed and optimized by TLL The Longevity Labs (Graz, Austria). The extraction method will obtain polyamines from wheat germs without the application of acids, organic solvents, and/or potentially harmful chemicals. Wheat germs serve as raw material for the extraction process, because they contain a high concentration of polyamines, in particular, spermidine [43].

The plant extract to be administered is mainly enriched by spermidine and spermine (with 1.2 mg spermidine and 0.6 mg spermine per 1 g extract). Furthermore, 1 g of extract contains 0.2 mg putrescine, < 0.005 mg cadaverine, and 0.166 mg l-ornithine. In combination with a normal diet, the intake of the planned daily dose of 750 mg extract (even in the case of multiple overdoses) is below the calculated no observed adverse effects level (NOAEL) for humans of 29 mg/kg body weight (bw)/day for cadaverine and putrescine [44]. This is also the case for spermidine and spermine, where the NOAEL is 13.5 mg/kg bw/day or 3.1 mg/kg bw/day [44]. The NOAEL for l-ornithine in humans is above 500 mg/kg bw/day [45], and thus remains unattainable in the planned extract administration. The safety of the spermidine-rich extract for the use in humans was evaluated prior to the SmartAge trial by a chemical analysis (unpublished data) and a translational study on safety and tolerability [36].

In the intervention group, the spermidine supplement will be administered daily in the form of six capsules, each containing 125 mg extract, resulting in a daily dose of 750 mg extract or 0.9 mg spermidine, 0.5 mg spermine, 0.2 mg putrescine, < 0.004 mg of cadaverine, and 0.12 mg of l-ornithine. This amount of daily polyamine intake accounts for an increase of approximately 10–20% of the average spermidine intake in industrial countries [46]. The dosage is within the amount of polyamines administered in an earlier intervention study [35], using approximately 10 mg/day of dietary spermidine in humans. The amount would also be obtainable by well-targeted diets (e.g., 200 g of cooked soybeans [47]). The control group will receive placebo capsules, six per day, filled with 750 mg cellulose in sum.

Participants of both groups (spermidine and placebo) are instructed to follow a regular capsule intake per day, two capsules with each main meal (breakfast, lunch, dinner), and to maintain their dietary habits during the time of intervention. Participants will be supplied with capsules throughout the intervention period. To ensure trial compliance, we closely monitor capsule intake throughout the trial (see the “Intervention period” section).

### Assessment of study measures

#### Measures assessed at baseline

Assessments of following participant’s characteristics will be conducted at baseline, summarized in Table 1: (a) *demographic information* including age, civil status, and education; (b) *information on family history* focused on AD, other non-specified subtypes of dementia, idiopathic Parkinson’s disease, and stroke; and (c) *behavioral measures* of subjective cognition function, lifelong experience, and personality traits. In addition, (d) *physiological measures* of brain Aβ status, measured using [18F] florbetaben (FBB) PET, and genotype information on apolipoprotein E (APOE) ε4 status along with other learning-relevant polymorphisms, measured using genotyping of blood-derived deoxyribonucleic acid (DNA), will be obtained. Note that potential changes in demographic information and family history will be recorded throughout the trial.

**Table 1:** SmartAge study outcome assessment

Time point	Measurement	Mode	V0	V1	V2	V3
Enrollment						
Eligibility screening		Telephone	x			
Screening assessment	MMSE [85]	On-site paper	x		x	x
	Logical Memory [53]	On-site paper	x		x	x
	TMT A [54]	On-site paper	x		x	x
	GDS [56, 86]	On-site paper	x		x	x
	IADL [55]	On-site paper	x		x	x
Signed informed consent		On-site	x			
Allocation/randomization				x		
Intervention						
Assessments						
Baseline variables						
Demographic information	Age, civil status, education	On-site paper		x		
Family history	Dementia, Parkinson disease, and stroke	On-site paper		x		
Subjective cognitive function	ECog-39 [87]	On-site paper		x		
Lifestyle (lifelong)	LEQ [88]	At-home paper		x		
	CAI [89]	On-site interview		x		
Personality	BFI-10 [90]	On-site paper		x		
	SVF-78 [91]	On-site paper		x		
Hand preference	Oldfield Hand Preference [92]	On-site paper		x		
Premorbid IQ	MWT [93]	On-site paper		x		
Genetic markers	APOE ε4 status	On-site		x		
Cerebral PET (optional consent)	Aβ status	On-site		x		
Primary outcome						
Memory	MST [38]	On-site Computer		x	x	x
Secondary outcomes						
Memory	VLMT [94]	On-site paper		x	x	x
	Doors and People [95]	On-site paper		x	x	x
Executive function	Digit Symbol [96]	On-site paper		x	x	x
	TMT B [54]	On-site paper		x	x	x
	Block Tapping [97]	On-site paper		x	x	x
	Stroop [98]	On-site paper		x	x	x
Attention	Digit Span [96]	On-site paper		x	x	x
	TAP [99] subtests: alertness and divided attention	On-site Computer		x	x	
Language	Semantic/Phonemic Fluency [100]	On-site		x	x	x
	Boston Naming Test [100]	On-site paper		x	x	x
Lifestyle (current)						
Physical activity	FKA [101]	At-home paper		x	x	x
Cognitive activity	CAI Present [89]	On-site interview		x	x	x
Sleep quality	PSQI [102]	At-home paper		x	x	x
Diet	FFQ [33, 63]	At-home paper		x	x	x
	MEDAS [103, 104]	On-site paper		x	x	x
	FFL [19, 62]	At-home paper		x	x	x
Psycho-affective/worry	PSWQ [105, 106]	At-home paper		x	x	x
	RSQ-D [107]	On-site paper		x	x	x
	STAI-G [108]	On-site paper		x	x	x
Quality of life	SF-12 [109]	On-site paper		x	x	x
	WHOQOL-BREF [110]	At-home paper		x	x	x
Autophagy markers from muscle biopsy (optional consent)	LC3 I/II, p62 [72], EP300 [111], proteomics, metabolomics [73]	On-site		x	x	
Blood-based markers	Polyamine levels [33], metabolomics, proinflammatory biomarkers, and neurotrophin levels	On-site		x	x	
Cerebral neuroimaging markers	Brain structure, perfusion, function	On-site		x	x	
Exploratory outcomes						
Subjective cognitive function	ECog-39, adapted [87]	On-site paper		x	x	x
	MMQ, adapted [112]	On-site paper		x	x	x
Cardiovascular risk factors	Blood pressure, lipid profile, glucose metabolism	On-site		x	x	x
Muscle function/strength*	SPPB [113]	On-site		x	x	x
	Handgrip strength	On-site		x	x	x

*Only for participants, who consented to muscle biopsy

*Abbreviations*: *Aβ* β-amyloid, *APOE* apolipoprotein E, *BFI-10* Big Five Inventory-10, *CAI* Cognitive Activity Interview, *ECog* Everyday Cognition Scales, *FKA* Freiburger Fragebogen zur körperlichen Aktivität, *FFL* food frequency list, *FFQ* Food Frequency Questionnaire, *GDS* Geriatric Depression Scale, *IADL* Instrumental Activities of Daily Living Scale, *LEQ* Lifetime Experience Questionnaire, *MEDAS* Mediterranean Diet Adherence Screener, *MMQ* Meta Memory Questionnaire, *MMSE* Mini-Mental State Examination, *MST* Mnemonic Similarity Task, *MWT* Mehrfachwahl-Wortschatztest, *PET* positron emission tomography, *PSQI* Pittsburgh Sleep Quality Index, *PSWQ* Penn State Worry Questionnaire, *RSQ-D* Response Styles Questionnaire—Deutsche Version, *SF-12* Short Form Health Survey, *SPPB* Short Physical Performance Battery, *STAI-G* State-Trait Anxiety Inventory, *SVF-78* Stressverarbeitungsfragebogen, *TAP* Testbatterie zur Aufmerksamkeitsprüfung, *TMT* Trail Making Test, *VLMT* Verbaler Lern-und Merkfähigkeitstest, *WHOQOL-BREF* World Health Organization Quality of Life

#### Outcome measures

Assessments of study outcomes will be conducted at the baseline visit and post-intervention visit (12 months after baseline). Selected outcome measures will be assessed at the follow-up visit (18 months after baseline). Primary and secondary outcomes are summarized in Table 1. Study outcomes will be collected in accordance with standardized operational procedures (SOPs) and study assessors that are blinded to the type of intervention.

Primary endpoint: The primary endpoint of this trial is the change in memory performance between baseline visit (V1) and post-intervention visit (V2). Memory performance is operationalized by mnemonic discrimination ability, to be assessed by the MST [38]. Mnemonic discrimination performance is evaluated due to its established sensitivity and robustness to memory deficits associated with aging and neurodegenerative disease [48–50]. Moreover, this behavioral marker is closely tied to neural dysfunction in the hippocampal memory network [48, 49] and has been identified as a sensitive outcome measure in older individuals at higher risk of AD [39].

The visual memory task is available for public download (http://faculty.sites.uci.edu/starklab/mnemonic-similarity-task-mst/) with multilingual instructions. Sets of well-matched stimuli will be presented at baseline, post-intervention, and follow-up visits respectively, with a pseudorandomized order of stimuli within each set. The MST consists of two phases: During the incidental encoding phase, participants view images of everyday objects and decide on each trial, whether the object is typically found “outdoors” or “indoors.” During the subsequent recognition phase, images are repeated (repetition items), randomly inter-mixed with novel images (foil items), and images that are perceptually similar to those pictures seen during the encoding phase (lure items). Participants will be asked to indicate for each trial, whether the image was “old”, “new” or “similar”. From the proportion of responses provided during recognition, a response bias-corrected mnemonic discrimination index will be calculated, similar to previous reports [37, 38, 48].

Secondary endpoints: Secondary outcomes include the change in memory performance (operationalized by mnemonic discrimination performance) between baseline visit (V1) and follow-up assessment (V3, 18 months after baseline). Additional secondary endpoints (see Table 1) are changes in the following outcome measures:

- *Neuropsychological parameters* on verbal and visual-spatial memory, attention, executive functions, and sensorimotor speed, assessed at V1, V2, and V3
- *Behavioral parameters* of lifestyle behaviors, psycho-affective characterization and perceived quality of life, assessed at V1, V2, and V3, as well as
- *Physiological parameters* including autophagy signaling (measured in muscle biopsies), peripheral vascular parameters (measured in blood), and parameters of brain structure, perfusion, and function (measured using cerebral magnetic resonance imaging [MRI]) to be assessed at V1 and V2.

Finally, exploratory outcomes (see Table 1) of subjective cognitive function, cardiovascular risk factors, as well as muscle function and strength markers (available for a sub-sample) will be evaluated.

Using moderator analysis, we will further assess whether demographic factors (including sex), genetic phenotype (including APOE), and presence of brain pathology (including a positive brain Aβ status) affect outcomes of the intervention.

### Participant timeline

**Fig. 1:**
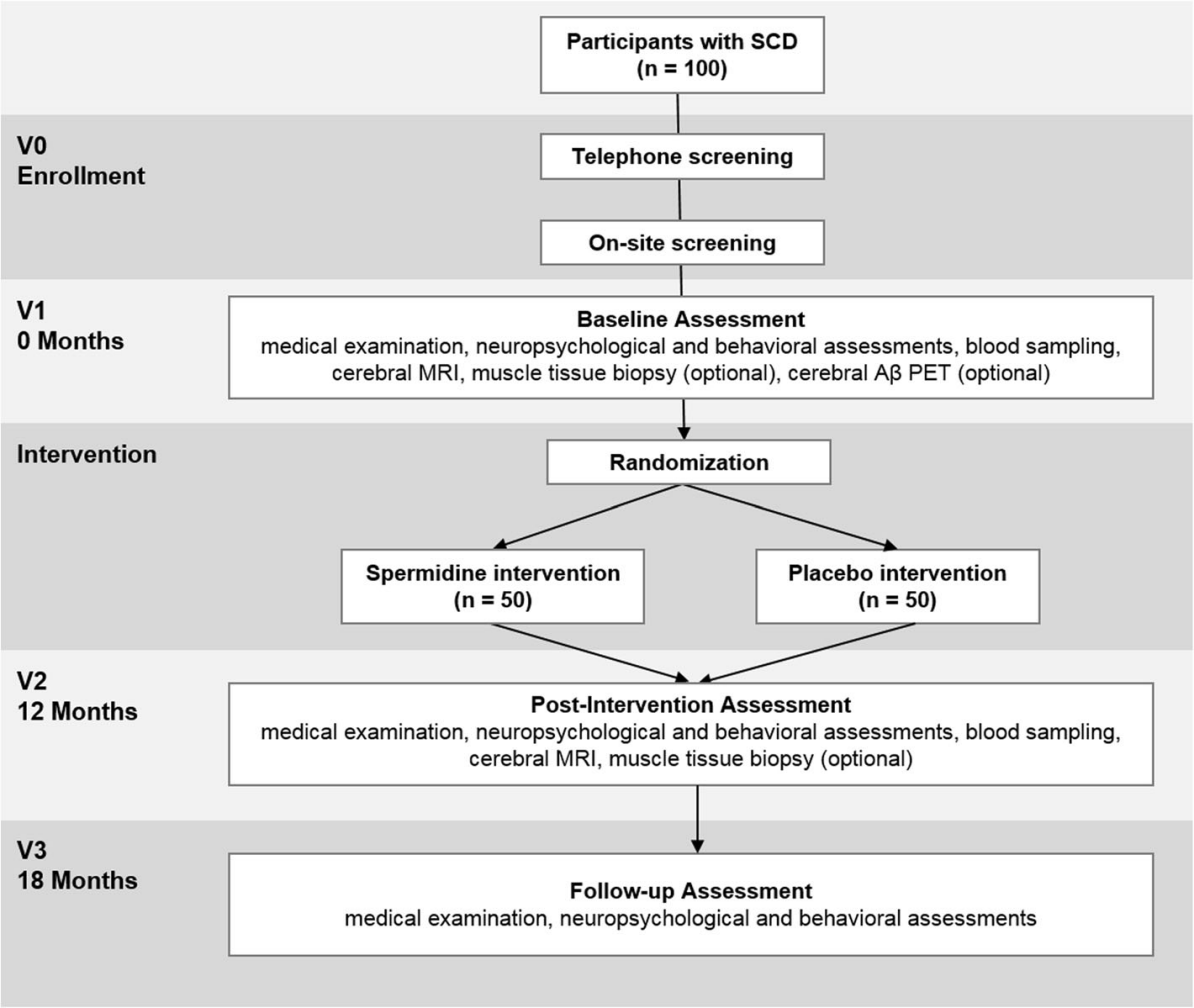
SmartAge study flowchart. Abbreviations: MRI: magnetic resonance imaging; *PET* positron emission tomography, *SCD* subjective cognitive decline

The SmartAge trial will involve five phases for each participant: study enrollment, which included screening assessment (V0), a baseline visit (V1), a 12-month intervention period, a post-intervention visit (V2), and a follow-up visit (V3). Trial phases are described below and summarized in Fig. 1.

#### Enrollment (V0)

Individuals, who express interest in study participation, will undergo following screening procedure to ensure study eligibility. A standardized pre-screening interview conducted over the phone will be administered to collect information on medical and demographic data, on MRI/PET suitability, and on suitability for muscle biopsy. During this interview, the following questions will be asked to endorse the presence of SCD with associated concerns (worries), similar to previous reports and recommendations [15, 51]. Question 1 will be “Do you feel that your cognitive performance has become worse?” (German: “Haben Sie das Gefühl, dass Ihre geistige Leistungsfähigkeit schlechter geworden ist?”, possible answers: yes/no). In case of a positive answer to question 1, question 2 will be asked: “Since when do you have the feeling that your cognitive performance has gotten worse?” (German: “Seit wann hat sich ihre geistige Leistungsfähigkeit verschlechtert?”). In case the answer to question 2 is at least 6 months, question 3 will be asked: “Are you concerned about this cognitive worsening?” (German: “Bereitet Ihnen diese Verschlechterung Sorgen?”, possible answers: yes/no). In case of a positive answer to question 3, question 4 will be asked: “Would you seek or have you sought medical help due to this cognitive worsening?” (German: “Würden Sie diesbezüglich einen Arzt aufsuchen bzw. haben Sie dies bereits getan?”, possible answers: yes/no). Affirmative responses to diagnostic questions 1, 3, 4, and a duration of SCD for at least 6 months are mandatory for study inclusion.

Potential participants that meet inclusion criteria as evaluated during pre-screening will be invited for the on-site screening visit to the Charité – Universitätsmedizin Berlin. Each individual will receive a standardized screening assessment, including neuropsychological tests and questionnaires, to certify the absence of objective cognitive impairment and current psychiatric disorder (e.g., depression). Following measures will be used: (a) Mini-Mental State Examination (MMSE) [52] score ≥ 26, (b) performance above − 1.5 standard deviation (SD) of age-adjusted norms in the Logical Memory II subscale total delayed recall (Story A and B) [53], and the Trail Making Test (TMT) A [54], (c) no deficits on selected items of the Instrumental Activities of Daily Living Scale (IADL) [55], and (d) a Geriatric Depression Scale (GDS) [56] score ≤ 10. After successful completion of the on-site screening assessment, eligible participants will proceed to baseline assessment.

#### Baseline visit (V1)

The baseline visit will encompass a maximum of 4 days with different assessments conducted on each day. On day 1 (carried out immediately after screening, duration: approx. 5 h with breaks), each participant will receive a standardized medical examination that includes fasting blood sampling and physical assessments. Next, a standardized neuropsychological test battery and a questionnaire battery will be administered (Table 1). On day 2, each participant will undergo the MRI assessment (duration: approx. 2 h). On day 3, muscle tissue biopsy will be carried out to determine autophagy signaling (optional consent, duration: approx. 2–3 h, aiming for 25 participants). On day 4, a cerebral Aβ PET assessment is performed (optional consent, baseline visit only, duration: approx. 2–3 h, aiming for 50 participants). After completion of the baseline visit, participants will be randomly assigned to one intervention arm. The Aβ PET measurements will in most cases be conducted after intervention has started, due to logistic reasons.

#### Intervention period

During the intervention, at 3 months, 6 months, and 9 months after the baseline visit (V1), telephone or on-site interviews will be scheduled with each participant to obtain information on participants’ physical and mental well-being, trial compliance, changes in dietary habits, and current medication intake. The study assessor will also ask for possible adverse events (AEs) and serious adverse events (SAEs). At 6 months after baseline, the remaining capsules will be counted to monitor adherence to capsule intake and new capsules for the next 6 months will be provided. Moreover, participants will be asked for their subjective estimation of compliance to intervention. At the post-intervention visit (V2), capsule counting procedures and subjective estimation of compliance will be repeated.

#### Post-intervention visit (V2)

The post-invention visit will take place at the end of the 12-month intervention period. Assessments of the post-intervention visit will be conducted on a maximum of 3 days, to be scheduled in close temporal proximity. On day 1, the standardized screening assessment (see Table 1) is re-administered to determine possible changes in the SCD diagnostic criteria, followed by the same assessments as for the baseline visit, that is, the standardized medical examination, the standardized neuropsychological test battery and questionnaire battery. In addition, participants will be asked to provide qualitative feedback on the intervention. On day 2, the MRI assessment will be carried out and on day 3, the muscle tissue biopsy will be obtained (optional consent).

#### Follow-up visit (V3)

The follow-up visit will take place 18 months after baseline visit. The visit will include following assessments: standardized screening assessment, standardized medical examination without blood draw, and adapted versions of the standardized neuropsychological test battery and questionnaire battery (see Table 1). The participants will be asked to provide qualitative feedback on the intervention.

### Sample size

A power calculation was conducted to estimate the number of participants required to detect a group difference in the primary outcome (i.e., mnemonic discrimination performance) at the end of the intervention. Sample size estimation was based on behavioral data obtained in a phase IIa pilot trial [37]. In this pre-study on 30 SCD participants (dropout: *n* = 2) and a 3-month intervention period, we determined an effect size of Cohen’s *d* = 0.65 on the group difference in change of mnemonic discrimination performance between baseline (V1) and the end of intervention (V2). Since this is a first estimate of the effect size based on a small sample, we based our sample size estimation for the present phase IIb trial on a smaller effect size of Cohen’s *d* = 0.5. To demonstrate a significant effect in the primary outcome, 50 participants per group (including a 10% dropout rate) need to be incorporated in the analysis with an unpaired-sample *t* test (two-sided significance level = 0.05, power 80%). Sample size estimation was conducted using a conservative approach [57] based on an unpaired-sample *t* test, even though the intended analysis of the primary outcome will be performed using analysis of covariance (ANCOVA) models. Case number estimation was compiled with the R package “pwr” [58].

### Recruitment

Study participants will be recruited from memory clinics, neurologists, and general practitioners in private practice in Berlin and from the general population in Germany through advertisements.

## Methods: assignment of intervention

### Allocation

Participants will be randomly assigned to the spermidine or the placebo group. A blockwise (block size of 6) randomization sequence, stratified by age (60–70, 70–80, and 80–90 years) and sex, will be generated using a computer-based algorithm (http://www.randomization.com/). Participant allocation will be performed at a 1:1 ratio by a study investigator without involvement in outcome assessments.

### Blinding

This trial will be double-blind, hence, participants and study assessors will remain blinded to group assignment until after the follow-up visit (V3; last participant, last visit). Efficacy of blinding from the participants’ perspective will be determined by asking each participant at post-intervention visit (V2) and follow-up visit (V3) to provide guesses of the assigned intervention condition.

## Methods: data collection, management, and analysis

### Data collection methods

Medical, neuropsychological, behavioral, and physiological data will be collected from each participant. Details on data collection procedures are provided below; time points of collection are detailed in Table 1. To ensure the standardization of data collection, all study assessors will undergo systematic training. Most data will first be recorded on paper (see Table 1) and will be entered into electronic records after each study visit with quality checks ongoing throughout the study. Quality control will be ensured through regular data monitoring.

#### Medical history and examination

Medical history assessment will include information on demographics, past and current medication intake, and family history. Subsequently, medical and neurological examinations will be conducted, including measurements of systolic and diastolic blood pressure and heart rate at rest. In addition, venous blood samples will be taken. In order to additionally test the influence of spermidine intervention on age-related sarcopenia, muscle function and muscle strength will be assessed (only of participants consented for the muscle biopsy).

#### Neuropsychological and behavioral batteries

The trial will include validated paper-and-pencil and computer-based neuropsychological tests as well as behavioral questionnaires (see Table 1). These instruments were selected based on their sensitivity and relevance in the study of aging and in correspondence with protocols of ongoing studies [59–61]. The neuropsychological test battery will include tests on learning and memory, executive functions, attention and working memory, as well as sensorimotor speed. The questionnaire battery will comprise measures of psycho-affective characteristics, lifestyle information (dietary habits, cognitive attainment, and physical activity), quality of life, and personality traits.

With regard to the nature of the trial, several questionnaires will be applied to collect nutritional information from each participant. Specifically, these measures will include the Food Frequency Questionnaire (FFQ), the Mediterranean Diet Adherence Screener (MEDAS), and a diet questionnaire that was previously adapted from a qualitative food frequency list (FFL) used in several German large-scale surveys [19, 62] (see Table 1). Validity and reproducibility of the FFQ are well established and based on the gold standard by Willett and colleagues [63]. The FFQ was recently applied in the study of Eisenberg and colleagues [33], indicating that this questionnaire provides meaningful estimations about the participant’s polyamine intake per day.

Questionnaires will be completed on-site and at home by each participant. Trained raters will evaluate neuropsychological tests and questionnaires in accordance with existing guidelines in test or questionnaire manuals. If available, German versions of questionnaires are included. Otherwise, German translations will be acquired or provided by the DELCODE study [59].

#### Blood sampling, genotyping, and pre-analytics

Fasting venous blood samples will be collected to analyze various blood parameters and to conduct genotyping. Labor Berlin Charité Vivantes GmbH will perform analyses of blood samples for safety parameters and research parameters, including glucose metabolism and lipid profile, in accordance with established SOPs. Polyamine levels (spermidine, spermine, and putrescine), metabolomics, proinflammatory biomarkers, and neurotrophin levels in whole blood, serum and plasma samples will be analyzed by the Institute of Molecular Biosciences, University of Graz, Austria. In addition, analysis of intracellular proteins with focus on nutrient signaling, metabolic regulation, and membrane-bound cellular markers of aging will be measured in peripheral blood mononuclear cells (PBMCs) extracted from whole blood [64]. In isolated leukocytes obtained from blood samples, acetylation levels and autophagy flux will be analyzed in collaboration with the INSERM U1138, Centre de Recherche des Cordeliers, Paris, France. Genetic analyses will be conducted by the Department of Psychiatry, Martin Luther University, Halle-Wittenberg, Germany.

Preprocessing and intermediate storage of blood samples will be performed at the Charité NeuroCure – Labs and at the NeuroCure Clinical Research Center, Charité – Universitätsmedizin Berlin. Blood samples for Labor Berlin Charité Vivantes GmbH will be shipped immediately. All other samples will be stored at − 80 °C or at 4 °C in phosphate-buffered saline and sodium azide until shipment for further processing and analyses. NeuroHub biomarker management platform and LabVantage software will guarantee standardized workflows, documentation, and monitoring of pre-analytics including processing as well as storage as described previously [36, 65].

#### Magnetic resonance imaging

The MRI examination will take place on a 3-T scanner (Tim Trio, Siemens, Erlangen, Germany) at the *Berlin* Center for Advanced Neuroimaging (*BCAN*, Charité – Universitätsmedizin Berlin)*.* The SmartAge MRI protocol will include structural and functional sequences (see Table 2). For structural imaging, a T1-weighted three-dimensional magnetization-prepared rapid acquisition gradient echo (MPRAGE) sequence, a Fluid Attenuated Inversion Recovery (FLAIR) sequence, Diffusion Tensor Imaging (DTI), and a high-resolution T2-weighted structural scan will be acquired perpendicular to the longitudinal axis of the hippocampus. The protocol will further include the following functional sequences: a blood-oxygen-level-dependent (BOLD) functional echo planar imaging (EPI), as assessed during rest (resting-state functional MRI) and during task (task-related functional MRI), and pseudo-continuous arterial spin labeling (pCASL) for cerebral blood flow (CBF) quantification during rest [66, 67].

**Table 2:** Neuroimaging data acquisition parameters

Sequence	Main parameters [orientation, TR/TE/TI, FOV, slices, voxel size]
Structural sequences	
T1 MPRAGE	Sagittal, TR/TE/TI = 1900/2.52/900 ms, 256 × 256 mm^2^, 192 slices, 1.0 × 1.0 × 1.0 mm^3^
FLAIR	Axial, TR/TE = 11,000/97 ms, 230 × 230 mm^2^, 50 slices, 1.2 × 1.2 × 2.5 mm^3^
DTI	Axial, TR/TE = 7500/86 ms, 220 × 220 mm^2^, 61 slices, 2.3 × 2.3 × 2.3 mm^3^, 64 directions (*b* = 1000)
High-resolution hippocampus TSE	Coronar, TR/TE = 8020/48 ms, 150 × 150mm^2^, 24 slices, 0.4 × 0.4 × 2.0 mm^3^
Functional sequences	
Resting-state fMRI	Axial, TR/TE = 2300/30 ms, 192 × 192 mm^2^, 34 slices, 3.0 × 3.0 × 4.0 mm^3^
Task-related fMRI	Axial, TR/TE = 2000/30 ms, 192 × 192 mm^2^, 32 slices, 3.0 × 3.0 × 3.0 mm^3^
pCASL	Axial, TR/TE = 4300/19 ms, 192 × 192 mm^2^, 22 slices, 3.0 × 3.0 × 5.0 mm^3^, LD/PLD = 1.5/1.5 s

*Abbreviations*: *DTI* diffusion tensor imaging, *FLAIR* fluid-attenuated inversion recovery, *fMRI* functional magnetic resonance imaging, *FOV* field of view, *LD* labeling duration, *MPRAGE* magnetization-prepared rapid acquisition gradient echo, *pCASL* pseudo-continuous arterial spin labeling, *PLD* post-label delay, *TE* echo time, *TI* inversion time, *TR* repetition time, *TSE* turbo spin echo

Functional MRI will include an associative face-name memory task, adapted and modified from a previous publication [68]. During the scanning session, the participants will learn 24 face-name pairs (encoding phase), which have to be correctly identified in a subsequent recognition phase. For details on this memory task, please refer to Sperling and colleagues [69, 70].

All MRI scans will undergo quality checks and will be evaluated quantitatively using state-of-the-art brain MRI software packages and toolboxes implemented in Statistical Parametric Mapping (SPM, Wellcome Department of Imaging Neuroscience), FSL (FMRIB Software Library, Oxford, UK), and FreeSurfer (Martinos Center for Biomedical Imaging, Massachusetts General Hospital, Boston, USA). Structural MRI scans will be used to examine the integrity of cerebral gray and white matter tissues, including assessments of volume, thickness, microstructure, and high-resolution volumetric quantification of medial-temporal structures [71]. Functional scans will be analyzed to assess brain activation patterns during the associative memory task as well as CBF maps and functional connectivity at rest.

#### Muscle tissue biopsy and muscle function (optional consent)

Muscle tissue biopsy will be performed at the Clinical Research Unit, Berlin Institute of Health (BIH, Charité – Universitätsmedizin Berlin). In total, 200–400 mg muscle tissue will be collected from the participant’s thigh (Musculus vastus lateralis). For this purpose, local anesthesia with lidocaine 1% without epinephrine will be administered. Subsequently, a skin incision (3–4 mm) will be made and the muscle tissue will be obtained by repeated needle biopsies (Bergström needle). Participants are asked to fast prior to the procedure for at least 8 h. After 12-month intervention time (V2) a second muscle tissue biopsy will be carried out at the same leg close to the first puncture site. Biopsy tissue processing will include snap freezing in liquid nitrogen and paraffin embedding. Samples will be stored at − 80 °C until analysis.

Potential changes in muscle autophagy markers in response to spermidine intervention will be quantified by immunohistochemical analysis of autophagic flux and autophagic capacity [72] as well as by acetylproteome analysis as an upstream regulator of autophagy [73].

In addition, non-invasive measurements of muscle function and muscle strength will be assessed during medical examination from all participants who consented to muscle biopsy, to determine age-related sarcopenia. For this purpose, the following standardized measurements of sarcopenia will be administered: Short Physical Performance Battery (SPPB) [74, 75], and a handgrip strength test [75]. The SPPB is a composite measure and will include 4-m gait speed, balance, and chair stand tests.

#### Positron emission tomography (optional consent)

Quantification of brain Aβ status is carried out using PET and the approved ligand FBB (Neuraceq™; Life Molecular Imaging (LMI)). The FBB PET images will be acquired using the PET/MR hybrid system (Siemens Biograph mMR) of the Institute of Diagnostic and Interventional Radiology and Nuclear Medicine, Charité – Universitätsmedizin Berlin. For data acquisition, 260–300 MBq of FBB PET tracer will be administered intravenously. Participants will undergo a static scan at 90–110 min after injection and images will be consistently reconstructed according to the current procedure guideline on brain PET imaging of the German Society of Nuclear Medicine [76].

The FBB PET scans will be processed using an automated pipeline based on the routines of the SPM software package, implemented in a MATLAB environment (Mathworks, Inc., Nattick, MA, USA). For each PET scan, intra-PET motion correction, construction of static standardized uptake value (SUV) images, and correction for partial volume effects using the Müller-Gärtner method [77] as implemented in the PETPVE12 toolbox [78] will be performed. Intensity scaling using the cerebellar cortex as reference region [79] will be applied to obtain standardized uptake value ratio (SUVr) images. Neocortical Aβ-plaques burden will be evaluated as the average FBB SUVr within a predefined composite of cortical regions-of-interest [76]. In addition, exploratory voxel-by-voxel analyses will be performed on the FBB SUVr images.

### Data management and monitoring

Documentation using a paper-based case report form (CRF) will be implemented. To certify excellent data quality, a good clinical practice (GCP) monitoring will be held on a regular basis. The SmartAge research team will be responsible for data management under the guidance of the principal investigator (AF). All CRF data will be entered into an electronic database with following quality control procedures: (a) double scoring and double entry of data points in the paper-based records and electronic records and (b) frequency distribution checks of outcome measures (not stratified by intervention group) to identify questionable data points.

### Adverse events monitoring and reporting

The occurrence of AEs and SAEs will be constantly monitored throughout the SmartAge trial. Participants will be instructed to immediately contact the research team in case of any self-noticed health changes or unexpected medical care visits. In addition, participants will receive a study pass, which informs about participation in the SmartAge trial and provides contact information in case of an SAE/hospitalization. All AEs and SAEs will be reported to and documented by the responsible study physician (SAEs within 24 h).

In general, the risk of health damage due to spermidine supplementation can be expected to be minimal, based on a prior interventional report [35] and our safety study [36]. Participants will be informed about all possible risks and can withdraw consent at any time without providing reasons. The expected “dropout” rate will be balanced in advance by the number of participants.

In case an SAE occurs, the study physician will first make an assessment as to whether or not a causal relationship with the intake of the investigational supplement is considered possible. Since this is a double-blinded trial, the assessment will be done without knowledge of group affiliation (spermidine and placebo). Emergency unblinding is possible, if the decision on follow-up medical treatment may depend on the participant’s allocated intervention during the trial. If more than three of the enrolled participants suffer from SAEs that are likely to be associated with spermidine intake (as assessed by the study physician), the SmartAge trial will be discontinued. All SAEs (whether or not related to the intervention) and all relevant pseudonymized documentation related to the SAE will be documented in the SAE report form, dated and signed by the principal investigator and included in the CRF documentation.

### Statistical methods

Statistical analyses of the primary outcome and secondary outcomes will be specified in the statistical analysis plan, to be written and registered before breaking the blind of study investigators. Statistical analyses of the primary outcome will be conducted by a designated statistician. Statistical analyses of secondary outcomes and additional exploratory analyses will be carried out by study investigators.

The primary outcome will be analyzed using an “intention to treat” (ITT) approach, consisting of all participants randomized into the trial, regardless of length of intervention. Subsequently, a “per protocol” analysis is carried out, including only those participants, who finished the 12-month intervention period. We will assess the between-group difference in the change of memory performance, operationalized by mnemonic discrimination performance, between baseline (V1) and the end of intervention (V2). Statistical analysis will be performed using an ANCOVA model with the change in memory performance (V2–V1) as dependent variable, intervention group as independent variable, and baseline memory performance as well as age as co-variates. Analyses of secondary endpoints will be conducted using comparable statistical methods. For example, analysis of change in memory performance between baseline (V1) and follow-up assessment (V3) will be carried out by means of ANCOVA. In this model, change in memory performance (V3–V1) will represent the dependent variable, with intervention group as the independent variable and baseline scores and age inserted as covariate.

In case of missing primary endpoints, the primary analysis will be done by using multiple imputation methods. It will further be investigated whether demographic or biological factors are associated with primary and secondary endpoints and / or modulate the response to the intervention, by integrating these variables into statistical models as covariates (or as interactive term with the intervention group). The main hypothesis is tested at a two-sided significance level of alpha = 0.05, using the ITT data set with multiple imputation.

### Dissemination policy

Results of the SmartAge trial will be distributed to scientific researchers and health-care professionals using peer reviewed journals and presentations (oral and written) at national and international scientific conferences. Publications will follow international recommendations of “Uniform Requirements for Manuscripts Submitted to Biomedical Journals” (http://www.icmje.org/recommendations/). Results will also be made available for scientific and lay audiences on the ClinicalTrials.gov website (Registered in ClinicalTrials.gov with the Identifier NCT03094546). In addition, we will transmit findings to the general population and key stakeholders using media coverage, such as newspaper articles and radio/television interviews.

## Discussion

This is the first randomized controlled phase IIb trial to determine potential beneficial impacts of a 12-month nutritional spermidine supplementation on memory performance in older individuals at risk for the development of AD. In addition, we will assess memory performance at 18 months and outcome measures of other neurocognitive domains as well as behavioral and physiological parameters to evaluate further benefits of the intervention.

The target group of the SmartAge trial comprises cognitively unimpaired older individuals with SCD. Given the fact that this population is at higher risk of objective cognitive decline and clinical progression [7, 8, 15], it is the key goal of this study to contribute to the development of effective prevention strategies. Our initial short-term (3 months) phase IIa trial demonstrated that spermidine supplementation is an easy-to-use, safe, and well-tolerated intervention [36]. Based on existing findings in animals and in humans [30, 32, 37], oral spermidine intake may be expected to protect memory performance and, as a consequence, favor perceived quality of life in older individuals with SCD.

There are several caveats that are important to acknowledge. Subjective cognitive decline is a heterogeneous condition that likely includes different etiologies as well as individuals, who will experience objective cognitive decline, while others remain stable for a long period of time. Although significant progression has been made to establish diagnostic criteria for SCD, there is no universal standard for the operationalization of SCD in clinical trials [80]. We have followed the existing guidelines and recommendations by the international SCD-I working group for studies on SCD [15] and examined other major SCD characterization cohort studies [81]. Likewise, cutoff scores for cognitive abilities are not strictly defined in the field, with cognitive deficits varying between 1 and 2 standard deviations below the normative mean [82]. Based on the literature review, we chose to use a 1.5 standard deviations cutoff to define cognitive abnormality, in line with other major SCD studies in the field [59]. Therefore, conceivably some of the recruited participants in this trial will be on the lower spectrum of cognitive abilities, close to mild cognitive impairment. Strengths of using a “more liberal” cognitive threshold include increased average rates of cognitive decline over the intervention period. We have further enriched our sample characterization through AD biomarkers, such as brain Aβ status (available for a sub-sample) and genetic risk factors. On the basis of this information, moderator analyses will be performed to estimate the effect of these AD variables on the intervention outcomes.

Other limitations of this phase IIb trial include the time period of intervention, which is still relatively short given the slow evolution of AD pathogenic processes in non-demented older individuals [83], and the restricted number of participants. Larger multi-center phase III trials with 1000–2000 participants and trial durations of 1.5–2 years commonly select clinical outcome measures, which are however less sensitive [84]. We deliberately chose a highly sensitive performance-based outcome measure, given our relatively short intervention time and manageable number of subjects. Although we recognize the importance of larger sample sizes, we remain optimistic that any subtle cognitive changes in our at-risk population will be detected based on our previous phase IIa trial [37]. Lastly, it is important to highlight that a multi-center trial implementation was not financially attainable. Pending the positive results of this trial, public funding for a phase III trial will be applied for, to move this intervention into clinical routine.

Overall, the SmartAge trial aims to contribute to the establishment of an effective and well-tolerable nutrition intervention to promote brain and cognitive health in older individuals at higher risk of dementia. A positive outcome with regard to memory performance in the spermidine-treated group may initiate a large multi-center phase III trial with a profound impact on public health, patients with SCD, and their families.

## Trial status

Recruitment of participants started in January 2017 and is expected to run until March 2019. The last follow-up is scheduled for September 2020.

## Abbreviations

AD: Alzheimer’s disease
AE: Adverse event
ANCOVA: Analysis of covariance
APOE: Apolipoprotein E
Aβ: β-Amyloid
BCAN: Berlin Center for Advanced Neuroimaging
BFI-10: Big five inventory-10
BOLD: Blood-oxygen-level-dependent
bw: Body weight
CAI: Cognitive Activity Interview
CBF: Cerebral blood flow
CRF: Case report form
DNA: Deoxyribonucleic acid
DSM-IV: Diagnostic and Statistical Manual of Mental Disorders, 4th Edition
DTI: Diffusion Tensor Imaging
ECog: Everyday Cognition Scales
EPI: Echo planar imaging
FBB: Florbetaben
FFL: Food frequency list
FFQ: Food Frequency Questionnaire
FKA: Freiburger Fragebogen zur körperlichen Aktivität
FLAIR: Fluid-attenuated inversion recovery
fMRI: Functional magnetic resonance imaging
GCP: Good clinical practice
GDS: Geriatric Depression Scale
IADL: Instrumental Activities of Daily Living Scale
ITT: Intention to treat
LEQ: Lifetime Experience Questionnaire
MCI: Mild cognitive impairment
MEDAS: Mediterranean Diet Adherence Screener
MMSE: Mini-Mental State Examination
MPRAGE: Magnetization-prepared rapid acquisition gradient echo
MRI: Magnetic resonance imaging
MST: Mnemonic similarity task
MWT: Mehrfachwahl-Wortschatztest
NOAEL: No observed adverse effects level
PBMCs: Peripheral blood mononuclear cells
pCASL: Pseudo-continuous arterial spin labeling
PET: Positron emission tomography
PSQI: Pittsburgh Sleep Quality Index
PSWQ: Penn State Worry Questionnaire
RSQ-D: Response Styles Questionnaire—Deutsche Version
SAE: Serious adverse event
SCD: Subjective cognitive decline
SD: Standard deviation
SF-12: Short Form Health Survey
SOP: Standardized operational procedure
SPM: Statistical Parametric Mapping
SPPB: Short Physical Performance Battery
STAI-G: State-Trait Anxiety Inventory
SUV: Standardized uptake value
SUVr: Standardized uptake value ratio
SVF-78: Stressverarbeitungsfragebogen
TMT: Trail Making Test
VLMT: Verbaler Lern-und Merkfähigkeitstest
WHOQOL: World Health Organization Quality of Life
