# Correction: Effects of spermidine supplementation on cognition and biomarkers in older adults with subjective cognitive decline (SmartAge)—study protocol for a randomized controlled trial

**DOI:** 10.1186/s13195-022-01012-9

**Published:** 2022-06-11

**Authors:** Miranka Wirth, Claudia Schwarz, Gloria Benson, Nora Horn, Ralph Buchert, Catharina Lange, Theresa Köbe, Stefan Hetzer, Marta Maglione, Eva Michael, Stefanie Märschenz, Knut Mai, Ute Kopp, Dietmar Schmitz, Ulrike Grittner, Stephan J. Sigrist, Slaven Stekovic, Frank Madeo, Agnes Flöel

**Affiliations:** 1 Charité – Universitätsmedizin Berlin, Corporate Member of Freie Universität Berlin, Humboldt-Universität zu Berlin, and Berlin Institute of Health, Klinik und Hochschulambulanz für Neurologie, Berlin, Germany; 2 Charité – Universitätsmedizin Berlin, Corporate Member of Freie Universität Berlin, Humboldt-Universität zu Berlin, and Berlin Institute of Health, NeuroCure Clinical Research Center, Berlin, Germany; 3 Charité – Universitätsmedizin Berlin, Corporate Member of Freie Universität Berlin, Humboldt-Universität zu Berlin, and Berlin Institute of Health, Center for Stroke Research Berlin, Berlin, Germany; 4 German Center for Neurodegenerative Diseases (DZNE), Dresden, Germany; 5 Charité - Universitätsmedizin Berlin, Corporate Member of Freie Universität Berlin, Humboldt-Universität zu Berlin, and Berlin Institute of Health, Department of Nuclear Medicine, Berlin, Germany; 6 University Hospital Hamburg-Eppendorf, Department of Diagnostic and Interventional Radiology and Nuclear Medicine, Hamburg, Germany; 7 Douglas Mental Health University Institute, Studies on Prevention of Alzheimer’s Disease (StOP-AD) Centre, Montreal, Quebec Canada; 8 Department of Psychiatry, McGill University, Montreal, Quebec Canada; 9 Charité – Universitätsmedizin Berlin, Corporate Member of Freie Universität Berlin, Humboldt-Universität zu Berlin, and Berlin Institute of Health, Berlin Center for Advanced Neuroimaging, Berlin, Germany; 10 Bernstein Center for Computational Neuroscience, Berlin, Germany; 11 Institute for Biology/Genetics, Freie Universität Berlin, Berlin, Germany; 12 Charité - Universitätsmedizin Berlin, Corporate Member of Freie Universität Berlin, Humboldt-Universität zu Berlin, and Berlin Institute of Health, Department of Endocrinology & Metabolism, Berlin, Germany; 13 Charité-Center for Cardiovascular Research (CCR), Berlin, Germany; 14 German Center for Neurodegenerative Diseases (DZNE), Berlin, Germany; 15 Charité – Universitätsmedizin Berlin, Corporate Member of Freie Universität Berlin, Humboldt-Universität zu Berlin, and Berlin Institute of Health, Institute of Biometry and Clinical Epidemiology, Berlin, Germany; 16 Berlin Institute of Health (BIH), Berlin, Germany; 17 Institute of Molecular Biosciences, University of Graz, NAWI Graz, Graz, Austria; 18 BioTechMed, Graz, Austria; 19 Department of Neurology, University Medicine Greifswald, Greifswald, Germany; 20 German Center for Neurodegenerative Diseases (DZNE), Rostock/Greifswald, Rostock, Germany

**Correction: Alz Res Therapy 11, 36 (2019)**

**https://doi.org/10.1186/s13195-019-0484-1**

Following the publication of the original article [1] the authors noticed a typo "Cohen's d = 0.5" on page 9 of the paper (subsection Sample size) and it should be corrected by "Cohen's d = 0.6".

The original article [1] has been updated.
